# Diabetes Mellitus, Energy Metabolism, and COVID-19

**DOI:** 10.1210/endrev/bnad032

**Published:** 2023-11-02

**Authors:** Caterina Conte, Elisa Cipponeri, Michael Roden

**Affiliations:** Department of Human Sciences and Promotion of the Quality of Life, San Raffaele Roma Open University, Rome 00166, Italy; Department of Endocrinology, Nutrition and Metabolic Diseases, IRCCS MultiMedica, Milan 20099, Italy; Department of Endocrinology, Nutrition and Metabolic Diseases, IRCCS MultiMedica, Milan 20099, Italy; Department of Endocrinology and Diabetology, Medical Faculty and University Hospital Düsseldorf, Heinrich-Heine-University Düsseldorf, Düsseldorf 40225, Germany; Institute for Clinical Diabetology, German Diabetes Center, Leibniz Center for Diabetes Research at Heinrich-Heine-University Düsseldorf, Düsseldorf 40225, Germany; German Center for Diabetes Research, Partner Düsseldorf, Neuherberg 85764, Germany

**Keywords:** SARS-CoV-2, adipose tissue, skeletal muscle, liver, pancreas, diabetes

## Abstract

Obesity, diabetes mellitus (mostly type 2), and COVID-19 show mutual interactions because they are not only risk factors for both acute and chronic COVID-19 manifestations, but also because COVID-19 alters energy metabolism. Such metabolic alterations can lead to dysglycemia and long-lasting effects. Thus, the COVID-19 pandemic has the potential for a further rise of the diabetes pandemic. This review outlines how preexisting metabolic alterations spanning from excess visceral adipose tissue to hyperglycemia and overt diabetes may exacerbate COVID-19 severity. We also summarize the different effects of SARS-CoV-2 infection on the key organs and tissues orchestrating energy metabolism, including adipose tissue, liver, skeletal muscle, and pancreas. Last, we provide an integrative view of the metabolic derangements that occur during COVID-19. Altogether, this review allows for better understanding of the metabolic derangements occurring when a fire starts from a small flame, and thereby help reducing the impact of the COVID-19 pandemic.

## Graphical Abstract

**Graphical Abstract:**
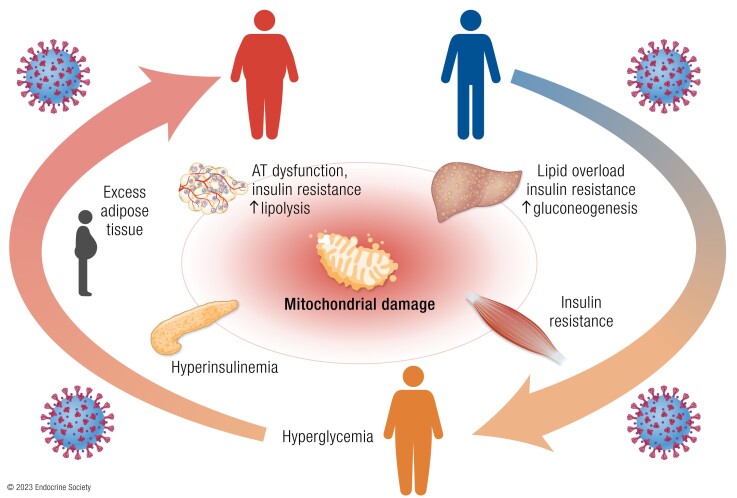

**Essential points**

- Preexisting obesity and diabetes increase vulnerability to SARS-CoV-2 infection and risk of severe COVID-19
- Both systemic inflammation and SARS-CoV-2-specific mechanisms lead to insulin resistance and can thereby contribute to hyperglycemia during acute COVID-19
- Hyperglycemia in people with COVID-19 is mainly driven by insulin resistance, which can persist even after clinical recovery
- Acute hyperglycemia, particularly in people without preexisting diabetes, is associated with worse outcomes in acute COVID-19

Since early 2020, SARS-CoV-2 has caused more than 761 million confirmed cases of the infectious COVID-19 and led to more than 6.8 million deaths globally (1). Although SARS-CoV-2 infection is asymptomatic or manifests with mild respiratory symptoms in most cases, some people develop critical illness, with acute respiratory distress syndrome (ARDS) requiring intensive care and then often leading to death. Older age, male sex, and obesity have been identified as the main risk factors for adverse outcomes in nearly all studies (2-6). Other chronic diseases, including diabetes mellitus, hypertension, and cardiovascular disease are also risk factors for severe COVID-19 and mortality (7-11). COVID-19 is often not limited to the acute manifestations because a variety of symptoms including respiratory impairment, generalized symptoms (eg, fatigue, joint pain, myalgia), psychiatric symptoms (eg, depression post-traumatic stress disorder), and neurological symptoms (eg, cognitive deficits, memory impairment) persist for months after recovery in approximately half of COVID-19 survivors (12). These post-COVID conditions, also known as post-acute sequelae of COVID-19 (PASC) or Long COVID syndrome, have been defined as the continuation or development of new symptoms 3 months after the initial SARS-CoV-2 infection, with these symptoms lasting for at least 2 months with no other explanation (13). PASC has been reported to affect up to 50% of people with COVID-19 (12, 14) and is more frequent in those with severe disease, but can affect any individual exposed to SARS-CoV-2 (15). Besides severe disease, older age and female sex are the main risk factors for PASC. Comorbidities including pulmonary disease, obesity, and diabetes also increase the risk (16-18). The relationship between obesity, diabetes (mainly type 2), and COVID-19 is bidirectional because not only are both conditions major risk factors for acute and chronic manifestations, but an association between COVID-19 and incident diabetes has also emerged (19-22).

Although the advent of effective vaccines has dramatically reduced COVID-19 morbidity and mortality (23) as well as the risk of PASC (24), COVID-19 remains prevalent and carries metabolic sequelae increasing incident diabetes (11). Given the impressive number of COVID-19 cases worldwide, the disease and its sequelae are a matter of public concern. Studies investigating the mechanisms by which metabolic alterations are a breeding ground for severe COVID-19, and how SARS-CoV-2 causes metabolic diseases, will allow us to understand the derangements that occur in energy metabolism when a fire breaks out from a small flame.

## COVID-19 and Metabolic Diseases: A Bidirectional Relationship

### Association of Metabolic Alterations With COVID-19 Severity and PASC

A link between severe forms of COVID-19 and metabolic alterations, including obesity and diabetes, emerged soon after the COVID-19 outbreak. Early studies reported an association of worse COVID-19 outcomes with obesity and other chronic conditions such as diabetes, hypertension, cardiovascular disease, and chronic kidney disease (25, 26). Subsequent studies found that an increased body mass index (BMI) is associated with the risk of invasive mechanical ventilation and severe COVID-19, and obesity, particularly class III obesity (BMI ≥ 40 kg/m^2^) is associated with increased mortality (27). The risk of hospitalization also increases with increasing BMI, particularly in younger individuals (<40 years) and in those of Black ethnicity (28). Of note, individuals with COVID-19 have been reported to have greater visceral adiposity than controls without COVID-19 but of similar age and BMI (29), and visceral but not subcutaneous adiposity is specifically associated with increased COVID-19 severity (30).

Diabetes, both type 1 and type 2, is associated with increased risks of COVID-19 infection and worse COVID-19 outcomes (7). Although individuals with worse glycemic control (glycated hemoglobin [HbA1c] ≥ 9% or 75 mmol/mol) had the highest risk of COVID-19 infection (∼60%), even those with adequate HbA1c levels (<7% or 53 mmol/mol) had a ∼20% greater risk compared with people without diabetes. Regardless of diabetes type, odds of hospitalization and worsening disease severity were more than 3-fold higher than in individuals without diabetes (8). A recent study estimated the global prevalence of diabetes according to COVID-19 severity (31). The prevalence of diabetes rose with increasing disease severity, from 10.4% (95% CI, 7.6-13.6) of nonhospitalized individuals to 21.4% (20.4-22.5) of hospitalized individuals. Among the latter, diabetes prevalence increased from 11.9% (10.2-13.7) in nonsevere COVID-19 to 28.9% (27.0-30.8) in severe COVID-19, and 34.6% (32.8-36.5) of deceased people with COVID-19.

Nonalcoholic fatty liver disease (NAFLD), defined by increased hepatic lipid accumulation in the absence of any other specific cause (32, 33), has been also associated with increased COVID severity, the risk of severe disease ranging from to 2.6- to more than 5-fold compared with individuals without fatty liver (34, 35). The risk of severe COVID-19 outcomes increases in near-linear manner with the number of components of the metabolic syndrome (impaired fasting glucose or diabetes, elevated blood pressure, hypertriglyceridemia, low high-density lipoprotein-cholesterol, or abdominal obesity), even after correction for BMI (36).

Obesity and diabetes also appear to increase the risk of PASC. A prospective observational cohort study of COVID-19 symptoms in more than 4000 adults found that the likelihood of persistent symptoms increased with increasing BMI (37). Several other studies have confirmed that obesity is a risk factor for PASC (38-40), the odds of developing persistent symptoms being 15% higher than in people without obesity (18). The risk of persistent symptoms is also elevated in individuals with preexisting diabetes (18, 41).

### Impact of SARS-CoV-2 Infection on Metabolic Diseases

COVID-19 survivors, even those who did not require hospitalization, are at higher risk of death and are more likely to use health care resources compared with people without COVID-19 because of a wide range of incident physical and psychological symptoms (42). The burden of post-acute sequelae is greater in individuals previously hospitalized for COVID-19 compared to those with seasonal influenza, suggesting disease-specific persistence of clinical manifestations (42). An excess burden of obesity, diabetes and hyperlipidemia has also been observed in people with a history of COVID-19 (42).

Similar to SARS-CoV (43, 44), COVID-19 can result in new-onset hyperglycemia or worsening glycemic control (45, 46), and new-onset type 2 diabetes is often listed among the long-term adverse consequences of COVID-19 (47). It has been estimated that the relative risk of diabetes in people with COVID-19 is 64% greater compared with non-COVID-19 controls, the association being stronger with type 2 diabetes (relative risk, 1.78; 95% CI, 1.56-2.02) than with type 1 diabetes (relative risk, 1.42; 95% CI, 1.38-1.46) (48). A study using electronic records of more than 850 000 individuals with COVID-19 and matched non-COVID-19 controls, who were followed up for 12 months after a diagnosis of COVID-19, found that the incidence of diabetes increased by 81% within 4 weeks of COVID-19 diagnosis and remained elevated by 27% from 4 to 12 weeks, returning to rates similar to those in the control group from 12 to 52 weeks (adjusted incidence rate ratio, 1.07; 95% CI, .99-1.16) (49). Other data support that the risk of developing diabetes is highest during the acute phase and only in those who were hospitalized for COVID-19, whereas it attenuates over time (50).

SARS-CoV-2 infection appears to promote the progression to diabetes in people with a history of prediabetes, but again only in individuals previously hospitalized for COVID-19 (51). Predictors of persistent diabetes at 5 months in those people comprise in-hospital diabetes diagnosis (hazard ratio [HR], 23.2; 95% CI, 16.1-33.4), critical illness (HR, 2.4; 95% CI, 1.6-3.8) and HbA1c (HR, 1.3; 95% CI, 1.1-1.4), but not SARS-CoV-2 infection (51). The latter, however, was a strong predictor of in-hospital diabetes (HR, 1.8; 95% CI, 1.4-2.3), along with critical illness (HR, 4.6; 95% CI, 3.5-6.1) and steroid treatment (HR, 2.88; 95% CI, 2.2-3.8).

Several studies have compared the risk of persistent diabetes following the acute phase in adults with COVID-19 vs adults with other respiratory illness (42, 52-58) (Table 1). The risk of type 2 diabetes increases with increasing severity of COVID-19, and in those with moderate/severe disease, this risk is 42% greater compared with moderate/severe influenza, even in persons not treated with steroid therapy (53). These estimates are based on a diagnosis of type 2 diabetes within 180 days after diagnosis of COVID-19 or influenza and might therefore include persons with previously undiagnosed diabetes. A more recent study found that the incidence of new-onset type 2 diabetes during hospitalization was 3.96 times higher (odds ratio, 3.96; 95% CI, 3.2-4.96) in persons with COVID-19 compared with those with influenza, but only 1.24 times higher (1.24; 1.07-1.45) at ∼3 months after diagnosis (21). Among people diagnosed with diabetes during hospitalization, 37% of those who returned for follow-up still had laboratory values indicating diabetes. Of those with no evidence of diabetes during hospitalization, 11.4% were diagnosed with type 2 diabetes at follow-up. Overall, the proportion of people with diabetes at ∼3 months after diagnosis was 16.7%. A diagnosis of type 2 diabetes during hospitalization, age, congestive heart failure, steroid therapy, intensive care unit admission, and D-dimer were significant predictors of diabetes at follow-up. Longer term studies confirm that glycemic control improves over time. Among people diagnosed with diabetes during hospitalization for COVID-19, 40.6% regressed to normoglycemia or prediabetes after a median of 323 (interquartile range [IQR], 205-385) days of follow-up. Most of those with persistent diabetes showed good glycemic control (HbA1c ≤ 7% or 48 mmol/mol) with diet and/or noninsulin medications, and only a minority of those discharged on insulin still required insulin at follow-up (59). Also, reinfection with SARS-CoV-2 is associated with higher diabetes risk (HR, 1.70; 95% CI, 1.41-2.05) (60). Anti-SARS-CoV-2 vaccination mitigates the risk of PASC but does not confer full protection in individuals with breakthrough infection, with the HR for metabolic disorders including diabetes and hyperlipidemia being 1.46 (1.37-1.56) compared with individuals without SARS-CoV-2 infection (52).

**Table 1.** Studies that compared the risk of diabetes in adults with COVID-19 vs adults with other respiratory illness

Author	Populations	Follow-up	Outcome	Risk
Al-Aly et al, 2021 (42)	COVID-19 (n = 13 654) Influenza (13 997)	COVID-19: 150 (84-217) d*^a^* Influenza: 157 (87-220) d*^a^*	DM—complication DM + complication T1D T2D DM, other*^c^*	HR (95% CI) 1.09 (.95-1.25) 1.11 (.96-1.28) .70 (.49-1.01) 1.14 (.96-1.34) .83 (.65-1.07)
Al-Aly et al, 2022 (52)	Breakthrough SARS-CoV-2 infection (n = 3667) Seasonal influenza (n = 14 337)	6 mo	DM Insulin use	HR (95% CI) 1.40 (1.05-1.87) 1.60 (1.26-2.05)
Birabaharan et al, 2022 (53)	COVID-19*^a^*: Mild (n = 313 924) Moderate/severe (n = 10 436) Influenza*^d^*: Mild (n = 319 783) Moderate/severe (n = 10 951)	6 mo	T2D	RR (95% CI) 1.54 (1.46-1.62) [mild] 1.22 (1.14-1.29) [mild, no steroids*^a,d^*] 1.46 (1.26-1.69) [moderate/severe] 1.42 (1.13-1.80) [moderate/severe, no steroids*^a,d^*)
Cohen et al, 2022 (54)	COVID-19 (n = 73,490, ≥ 65 y) VLRTI (n = 73,490, ≥ 65 y)	68 (23-155) d*^a^*	T2D	HR (95% CI) 1.23 (1.02-1.48)
Daugherty et al, 2021 (55)	COVID-19 (n = 181,613, 18-65 y) VLRTI (n = 181,613, 18-65 y)	87 (45-124) d*^a^*	T2D	HR (95% CI) 1.39 (1.22-1.58)
Holman et al, 2023 (56)	COVID-19, no pneumonia (n = 58 091) COVID-19 pneumonia (n = 29 006) non-COVID-19 pneumonia, year: 2020 (n = 99 951), 2019 (n = 117 148), 2018 (n = 110 489) 2017 (n = 102 733)	≥2 wk after hospital discharge	DM	IR (95% CI) 16.4 (12.8-20.7) [COVID-19 no pneumonia, reference] 19.0 (13.8-25.6) [COVID-19 pneumonia] Non-COVID pneumonia: 16.6 (13.3-20.4) [2020] 13.7 (10.8-17.3) [2019] 13.8 (10.9-17.4) [2018] 14.2 (10.9-18.3) [2017]
Lu et al, 2023 (21)	COVID-19 (n = 4982) Influenza (n = 2988)	COVID-19: 83 d*^b^* Influenza: 87 days*^b^*	T2D	OR (95% CI) 1.24 (1.07-1.45)
OpenSafely Collaborative, 2022 (57)	COVID-19 (n = 77 347) vs non-COVID-19 pneumonia (n = 127 987)	0–≥120 d	T2D	SHR (95% CI) 1.30 (1.15-1.47)
Rathmann et al, 2022 (58)	COVID-19 (n = 35 865) AURI (n = 35 865)	COVID-19: 119 (0-210) d*^a^* AURI: 161 (4-225) d*^a^*	T2D DM, other*^c^*	IRR (95% CI) 1.28 (1.05-1.57) 1.17 (.80-1.71)

Abbreviations: AURI, acute upper respiratory tract infections; DM, diabetes mellitus; HR, hazard ratio; IR, incidence rate per 1000 person-years; IQR, interquartile range; IRR, incidence rate ratio; OR, odds ratio; RR, relative risk; SHR, subdistribution hazard ratios; VLRTI, viral lower respiratory tract illness.

Follow-up data are *^a^*days, median (IQR) or *^b^*days, mean.

^*c*^Because of underlying condition, drug or chemical induced, or other specified type; *^a^*276 748 (88%) of those with mild COVID-19 and 5357 (51%) of those with moderate/severe COVID-19 were not treated with steroids

^*d*^280 851 (88%) and 5424 (50%) of those with moderate/severe influenza were not treated with steroids

^*^No significant difference between COVID-19 without pneumonia and other groups.

Despite the possibility that some people had previously undiagnosed diabetes before developing COVID-19 and that individuals who had COVID-19 undergo more intensive screening for medical conditions compared with uninfected people (61), there is now a large body of evidence that COVID-19 is associated with an increased diabetes risk, which is highest during the acute phase of COVID-19 (19, 21). Considering the large number of individuals affected by COVID-19 worldwide, these rates appear worrisome and suggest that the COVID-19 pandemic will cause a dramatic surge in the future number of people with diabetes globally.

## Mechanisms by Which Preexisting Metabolic Alterations may Increase COVID-19 Severity

SARS-CoV-2 enters cells via binding of the transmembrane spike glycoprotein to the angiotensin-converting enzyme-related carboxypeptidase (ACE2) receptor (62), a type I integral membrane peptidase that cleaves angiotensin II into angiotensin 1-7, a peptide with protective effects on several organs and systems (63). The virus is then either (1) internalized via clathrin-mediated endocytosis and the spike protein is cleaved by cathepsins, which prompts fusion of the viral and endosome membranes to release viral RNA into the cytosol, or (2) undergoes cleavage of the spike protein by the transmembrane protease serine 2 (TMPRSS2) on the cell surface, which allows membrane fusion and penetration of viral RNA into the cytosol (64). Other host factors such as neuropilin 1 (NRP1) and furin are involved in SARS-CoV-2 entry or spike protein activation (64). SARS-CoV-2 infection is asymptomatic or causes mild, flu-like symptoms in most cases. Few people develop the so-called “cytokine storm” (ie, a systemic uncontrolled, hyperinflammatory response characterized by the release of proinflammatory cytokines such as IL-6 that underlies the pathogenesis of ARDS, multiorgan failure and death) (65, 66). The following paragraphs will address the mechanisms by which preexisting metabolic alterations may increase COVID-19 severity.

### Obesity and Excess Visceral Adipose Tissue

#### Adipose tissue dysfunction and metabolic diseases

Obesity is a chronic relapsing progressive disease (67) characterized by abnormal fat mass and distribution and associated with several diseases including cardiovascular disease, diabetes, dyslipidemia and NAFLD (68). Over time, the excessive fat accumulation leads to abnormal expansion of white adipose tissue with adipocyte hypertrophy (enlarged cells) and hyperplasia (increased number of new smaller cells) (69, 70), which varies individually and depends on the respective adipose tissue compartment. Adipose tissue expansion is paralleled by local angiogenesis to supply nutrients and oxygen to adipocytes. The sequence of events seems to be hypertrophy followed by angiogenesis and hyperplasia favoring local hypoxia and adipose tissue dysfunction (71). Specifically, visceral adipose tissue exhibits lower angiogenic capacity with higher risk of hypoxia (72). Adipocyte hypoxia triggers local inflammation with release of chemotactic signals and proinflammatory cytokines and subsequent recruitment of macrophages with altered polarization, ultimately leading to fibrosis and adipocyte death (73-80). Recent studies in a high-fat diet model reported that the activation of *sn*-1,2-diacylglycerol/protein kinase Cε/insulin receptor threonine residue 1160 phosphorylation pathway represents a very early abnormality an of white adipose tissue (81). Activation of this pathway impairs insulin signaling and causes insulin resistance with impaired insulin-induced suppression of white adipose tissue lipolysis. This causes lipid overflow to other organs, triggering a cascade of events that eventually results in increased gluconeogenesis and fasting glucose production, and ectopic fat accumulation leading to lipotoxicity and multiorgan insulin resistance (82). Mechanical stress on the adipocyte, release of damage-associated molecular proteins from dying adipocytes, activation of inflammatory signaling by free fatty acids (FFA) through the Toll-like receptor 4 or Toll-like receptor 2 on the adipocyte's plasma membrane, endoplasmic reticulum (ER), and oxidative stress also contribute to adipose tissue inflammation and insulin resistance (83-85). These early mechanisms contribute to the onset of a local inflammation that over time leads to spillover of pro-inflammatory cytokines and adipokines into the systemic circulation, worsening the multiorgan insulin resistance triggered by excess release of lipids from adipose tissue (82). Adipose tissue macrophages are the main source of adipose tissue-derived pro-inflammatory cytokines and play a key role in the development of systemic inflammation, insulin resistance, and type 2 diabetes (79, 86). Excess adiposity is characterized not only by the infiltration of macrophages in adipose tissue, but also by a shift in their phenotype, from the “alternatively activated macrophages” phenotype (M2-like), which produces anti-inflammatory cytokines (eg, IL-10) to the “classically activated macrophages” phenotype (M1-like), which secretes pro-inflammatory cytokines (eg, IL-1, IL-6, TNF-α) (87). Besides proinflammatory macrophages, the adipose tissue in obesity is characterized by the recruitment and expansion of neutrophils, B cells, CD8^+^ T cells, and T helper 1 (T_H_1) cells, and by decreased eosinophils, natural killer T cells, type 2 innate lymphoid cells, and regulatory T cells (88). Eventually, the changes induced by excess adiposity cause local and systemic maladaptive, chronic low-grade inflammation, contributing to systemic insulin resistance (89-93). To overcome insulin resistance, β cells secrete a greater amount of insulin leading to hyperinsulinemia, which stimulates further expansion and dysfunction of adipose tissue. Over time, if excess adiposity is maintained, these alterations persist and β-cell function declines, causing fasting and postprandial hyperglycemia, and overt diabetes (82).

Longstanding adipose tissue dysfunction leads to altered expression and secretion of adipose-tissue specific cytokines (adipokines) involved in the regulation of energy metabolism, appetite regulation, and proinflammatory and immune responses. A lower expression of anti-inflammatory adipokines (eg, adiponectin) and higher expression of proinflammatory adipokines (eg, leptin, resistin, visfatin) contributes to the systemic subclinical inflammation and progressive insulin resistance with increased risk of type 2 diabetes (82). Alterations in the secretion of adipokines also play a role in obesity-related immune dysfunction. The altered metabolic milieu in obesity and/or type 2 diabetes may affect the metabolism of effector, regulatory, or memory cells. Immune cells are especially sensitive to changes in the lymphocyte microenvironment because they depend on extracellular nutrient uptake for survival and functioning (94). The IL-6-like adipokine leptin, whose levels increase proportionally to fat mass (95), is involved in both innate and adaptive immune responses (96). Leptin increases the number of T_H_1 and T_H_17 cells in both mice and humans and is necessary for glucose uptake via the glucose transporter GLUT1 of activated effector T cells, thereby allowing energy-dependent processes such as T-cell proliferation and cytokine production (97). Hyperleptinemia in obesity is therefore thought to contribute to altered immunity by affecting immune cell metabolism (98). The role of adiponectin in immunity has been explored less than that of leptin. Adiponectin exerts insulin sensitizing and anti-inflammatory effects and is reduced in obesity (99).

Finally, although the abundance of mitochondria in adipocytes is lower compared with other cell types, these organelles play an important role in energy homeostasis in adipose tissue, being key to metabolic processes such as adipogenesis, lipogenesis, fatty acid esterification, and lipolysis (100). Obesity is associated with abnormal mitochondrial function in adipose tissue, with reduced mitochondrial biogenesis, downregulation of genes encoding for components of the mitochondrial respiratory complex, and decreased oxidative phosphorylation (101-103). These alterations may contribute to, and further aggravate, adipose tissue inflammation and insulin resistance in obesity (104).

#### Adipose tissue and SARS-CoV-2

Several studies have demonstrated that SARS-CoV-2 can infect adipocytes, and that obesity and diabetes enhance the expression of SARS-CoV-2 entry factors in adipose tissue (105-109). Adipocytes express the SARS-CoV-2 entry factors ACE2, TMPRS2, transferrin receptor, NRP1, and Furin (107). ACE2 is highly expressed in adipose tissue, and its expression is amplified in obesity and obesity-related NAFLD (105, 106). ACE2 expression is greater in visceral adipocytes, which are more susceptible to SARS-CoV-2 infection than subcutaneous adipocytes (108). Additionally, following SARS-CoV-2 infection, visceral adipocytes show significantly higher expression of proinflammatory genes such as *TNFA* and *IL6* compared with subcutaneous adipocytes (108). A Mendelian randomization analysis confirmed that adiposity, particularly central adiposity (defined by increased waist-to-hip ratio), associates with risk of COVID-19 susceptibility, hospitalization, and disease severity, which is mediated at least partly by plasma ACE2 (110). The glucose-regulated protein 78 (GRP78) is an ER-associated protein that is translocated to the cell surface under stress conditions, and is highly expressed in adipose tissue, particularly visceral adipose tissue (109). GRP78 facilitates the binding of the spike protein to the ACE2 receptor (109, 111) and is upregulated by aging, obesity, diabetes, and hyperinsulinemia (109).

Thus, excess visceral adipose tissue in obesity could serve as a reservoir for SARS-CoV-2. A role in the onset of systemic, uncontrolled inflammation in COVID-19 can be hypothesized for several mechanisms involved in insulin resistance and inflammation in adipose tissue. Obesity-related chronic, low-grade systemic inflammation affects immune function and the lung's response to acute infection (112). Elevated levels of pro-inflammatory cytokines, both systemically and in the lungs, favor the development of more severe lung damage in response to infection (113, 114). Type I interferons (IFNs, mainly IFN-α and IFN-β), play a key role in the innate immune response to viral infections, including SARS-CoV-2 infection (115). The stimulator of interferon genes (STING) pathway mediates type I interferon (IFN) inflammatory responses in immune cells and is involved in the immune response for various respiratory inflammatory conditions. STING is considered to be a molecular connection between immunity and metabolism (116). Activation of STING in obesity increases the pro-inflammatory capacity of lung macrophages, a potential mechanism underlying obesity-related lung inflammation (117). Notably, STING is a major contributor to the aberrant type I IFN response in COVID-19, which amplifies the inflammatory response during the late phase of SARS-CoV-2 infection (118).

B cells expressing T-bet (T-bet^+^), a transcription factor unique to the T_H_1 lineage, accumulate in mouse and human adipose tissue during obesity. On activation, T-bet^+^ secrete the proinflammatory C-X-C motif chemokine 10/interferon-gamma-induced protein 10 (CXCL10), which exacerbates adipose tissue inflammation and glucose intolerance in obesity (119-121) and plays a key role in the pathogenesis of infections by human coronaviruses, having emerged as a prognostic marker for disease severity in COVID-19 (122). The NOD-like receptor pyrin-domain containing 3 (NLRP3) inflammasome activation in adipose tissue mediates reactive oxygen species production, release of IL-1β and IL-18, and promotion of systemic inflammatory reaction, possibly facilitating the “cytokine storm” (123-125). Of note, infection of mice with murine beta coronavirus A59 increases NLRP3 inflammasome-mediated inflammation (126).

Endothelial damage is another prominent feature of COVID-19. Binding of SARS-CoV-2 to the ACE2 receptor reduces ACE2 and the conversion of angiotensin II to angiotensin 1-7, thereby increasing the levels of angiotensin II (127), which has detrimental effects on glucose metabolism (128). On SARS-CoV-2 infection, elevation of angiotensin II levels and the increased release of the proinflammatory cytokines IL-1 and IL-6 induce endothelial activation and increase vascular permeability and expression of adhesion molecules, contributing to the development of a prothrombotic phenotype (129). Circulating levels of plasminogen activator-1, which inhibits plasma fibrinolytic activity (130) and is secreted by several cell types including visceral fat adipocytes and macrophages (131), are elevated in obesity and associate with detrimental metabolic and prothrombotic effects (132, 133). Plasminogen activator-1 is elevated in individuals with severe COVID-19 (107, 134), possibly contributing to the increased risk of venous thrombosis seen in people with obesity and COVID-19 (135). In addition, obesity-induced endothelial dysfunction because of adipose tissue-derived endocrine and paracrine signals such as adipokines and extracellular vesicles carrying bioactive molecules causes endothelial dysfunction (136), thereby further worsening SARS-CoV-2-induced endothelial damage.

White adipose tissue adipocytes are a major source of the tryptophan-derived metabolites, collectively known as kynurenines (137). Tryptophan is an essential amino acid that serves as a substrate for protein and serotonin synthesis, although only 5% of the bioavailable tryptophane is used in these processes (138). Most tryptophan is catabolized via the kynurenine pathway, which yields biologically active kynurenines such as quinolinic acid, kynurenic acid, and kynurenine, and the nicotinamide adenine dinucleotide, a cofactor in mitochondrial energy production and several enzymatic redox reactions (138, 139). Adults with overweight/obesity or type 2 diabetes have significantly increased serum kynurenine levels and kynurenine to tryptophane ratios (140, 141). This is mainly because of reduced conversion of kynurenine to kynurenic acid, and contributes to impaired lipid homeostasis and insulin sensitivity in adipocytes (137). Infections and inflammation are other conditions in which there is an enhancement of the kynurenine pathway. In fact, proinflammatory cytokines shift the metabolism of tryptophan toward the kynurenine pathway aiming to provide energy (nicotinamide adenine dinucleotide) to activated immune cells (142). Nevertheless, kynurenines also have an immunosuppressive effect, which is needed to resolve the inflammatory response (142). A preexisting elevation of kynurenine in individuals with obesity or type 2 diabetes likely contributes to the increased kynurenine levels and kynurenine to tryptophane ratio that has been reported in severe COVID-19 (143). Of note, activation of the kynurenine pathway in individuals with COVID-19 correlates with high IL-6 levels and, by blunting the immune response, may facilitate SARS-CoV-2 infection and the development of severe disease (144, 145).

### Type 2 Diabetes and Hyperglycemia

People with diabetes generally develop more severe COVID-19 than those without diabetes, showing a higher incidence of lymphopenia and increased biomarkers of inflammation (146). Type 2 diabetes is a proinflammatory condition (147), characterized by higher levels of IL-6, IL-8, and TNF-α, which are associated with worse clinical outcomes and are highest in the presence of comorbidities (148). Individuals with COVID-19 and type 2 diabetes were reported to have significantly greater levels of T_H_1 cytokines such as IFN-γ, and IL-6 compared with individuals with COVID-19, but not diabetes (149, 150).

The presence of diabetes or hyperglycemia due to other causes can affect COVID-19 clinical outcomes in multiple ways. Having diabetes may increase the susceptibility to SARS-CoV-2 infection. Studies in rodent models indicate that the expression of ACE2 is increased in diabetes (151, 152), and particularly type 2 diabetes was shown to be associated with increased *ACE2* expression (153, 154). Hyperglycemia induces glycation of several proteins, including ACE2, which either facilitates or reduces binding of SARS-CoV-2 to ACE2, depending on the site of glycation (155, 156). Furthermore, increased levels of TMPRSS2 (157) and furin (158) have been reported in hyperglycemia and diabetes. Individuals with diabetes have impaired innate immunity, including blunted neutrophil and macrophage chemotaxis and function, as well as impaired adaptive immunity and altered cytokine secretion. In mice infected with Middle East respiratory syndrome coronavirus, mice with high-fat diet-induced diabetes developed more severe disease and exhibited a dysregulated immune response, with decreased CD4^+^ T-cell and inflammatory monocyte/macrophage response and lower cytokine expression at the early stages of infection, indicating delayed initiation of inflammation (159). These derangements in host cellular responses may contribute to their increased risk to develop severe COVID-19. Consistently, poorer glycemic control in people with type 2 diabetes has been linked to greater mortality compared with better glycemic control (146), likely via hyperglycemia-mediated inhibition of lymphocyte proliferation (160).

In-hospital hyperglycemia, regardless of the presence of diabetes, is common and is associated with poor clinical outcomes not only in COVID-19, but also in a range of different clinical conditions (161-165). The impact on clinical outcomes is generally greater in individuals with new-onset hyperglycemia compared with those with preexisting diabetes (161, 166), and COVID-19 is no exception (167, 168). This increased susceptibility to glucose toxicity in people without preexisting diabetes is possibly from a lack of adaptive mechanisms, leading to cellular glucose overload and increased generation of and/or deficient scavenging of reactive oxygen species produced by activated glycolysis and oxidative phosphorylation (169). An impairment in the innate immune responses caused by acute hyperglycemia may also contribute (170). Hyperglycemia was also shown to enhance SARS-CoV-2 replication and the production of proinflammatory cytokines such as TNF-α and IL-6 in monocytes in vitro (171). Thus, preexisting diabetes may increase vulnerability to SARS-CoV-2 infection (7, 172) and the risk of unfavorable clinical outcomes (8). Although worse glycemic control is associated with worse outcomes (146), even individuals with diabetes and adequate glycemic control are at ∼45% greater risk of adverse outcomes compared with those without diabetes (7). This suggests that diabetes per se renders individuals more prone to severe COVID-19 compared with normoglycemic individuals without diabetes. Acute hyperglycemia, particularly in those without pre-existing diabetes, may act through different pathways, contributing to worse outcomes.

## Effect of SARS-CoV-2 Infection on Tissue-specific Energy Metabolism

This section examines the effects of SARS-CoV-2 infection on the main organs orchestrating glucose metabolism to better elucidate the relationship between COVID-19 and disrupted glucose metabolism.

### Adipose Tissue

**Figure 1.**
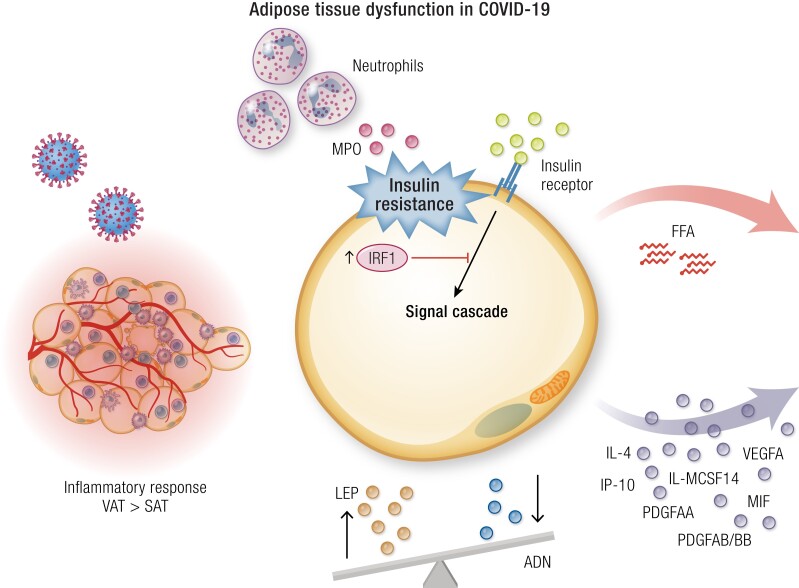
COVID-19 and the adipose tissue (see also Table 2). Abbreviations: ADN, adiponectin; FFA, free fatty acids; IP-10, interferon-γ–induced protein-10; IRF-1, interferon regulatory factor 1; LEP, leptin; MCSF1, macrophage colony-stimulating factor 1; MIF, macrophage migration inhibitory factor; MPO, myeloperoxidase; PDGFAA, platelet-derived growth factor AA; PDGFAB/BB, platelet-derived growth factor AB/BB; SAT, subcutaneous adipose tissue; VAT, visceral adipose tissue; VEGFA, vascular endothelial growth factor A.

SARS-CoV-2 has the ability to infect human adipose tissue (Table 2), targeting both mature adipocytes and a subset of inflammatory adipose tissue-resident macrophages and stromal vascular cells, leading to immune activation and the secretion of inflammatory factors associated with severe COVID-19, including interferon-γ-induced protein-10 (IP-10), platelet-derived growth factor AA, platelet-derived growth factor AB/BB, IL-4, macrophage migration inhibitory factor, vascular endothelial growth factor A, and macrophage colony-stimulating factor 1 (174) (Fig. 1). The inflammatory and antiviral response to SARS-CoV-2 infection is more prominent in visceral than subcutaneous fat (107) (Fig. 1), and alterations in visceral adipose tissue are more persistent in old compared with young hamsters infected with the virus (176). Preclinical studies have shown that SARS-CoV-2 infection induces adipocyte dysfunction (Fig. 1), causing a reduction in adiponectin expression that may contribute to systemic insulin resistance in individuals with severe COVID-19 (107). Reduced adiponectin is a hallmark of obesity, particularly central obesity (177). Low circulating adiponectin levels have been reported in people with COVID-19 compared with healthy controls (107, 178, 179), the reduction generally being greater with increasing disease severity (178, 180). Nevertheless, it is difficult to establish whether the reduction in adiponectin is COVID-19-specific because (1) low adiponectin levels have been reported in critical illness from other causes (181), (2) only some (107, 182), but not all (178, 183) studies demonstrated greater reductions of adiponectin in people with acute respiratory failure from COVID-19 compared with acute respiratory failure from other causes, and (3) decreased adiponectin has been reported with intensive insulin treatment aiming at near-normoglycemia compared with insulin administered only if blood glucose values were >220 mg/dL in critically ill patients (184). Similarly, leptin levels are increased (179, 185-187), unaffected (183, 188, 189), or even reduced (178). Of note, lower adiponectin-to-leptin ratio, a surrogate of adipocyte function (190), showed an association with worse COVID-19 outcomes (107, 178, 180, 185, 189).

**Table 2.** Main preclinical studies assessing the effect of SARS-CoV-2 infection on adipose tissue

Author	Model	Main findings
Reiterer et al, 2021 (107)	Golden hamsters Primary human adipocytes	↓ adiponectin gene expression (*Adipoq*) in SAT, not in VAT ↓ adiponectin protein levels in SAT, VAT, and serum (hamsters) Antiviral response*^a^*: VAT > SAT Adipocytes express *Ace2*, *Tmprss2*, *Nrp1*, *Furin*, *Trfc* Adipocytes permissive*^b^* to viral infection
Shin et al, 2021 (109)	Transcriptome data sets of human and mouse tissues 3T3-L1 adipocytes differentiated into mature adipocytes Murine adipose tissue	Expression of the *Grp78* gene: VAT > SAT ↑ in aging (VAT > SAT) ↑ in obesity and diabetes ↑ with chronic insulin exposure in adipocytes in part mediated by XBP-1s
Shin et al 2022 (173)	transcriptome datasets of SARS-CoV-2-infected cells and tissues	↑ IRF1 ↓ insulin/IGF pathway
Martinez-Colon et al, 2022 (174)	Human autopsies Adipocytes and ATMs from human EAT, VAT, SAT	SARS-CoV-2 RNA in EAT, VAT, and SAT of individuals with COVID-19 Mature adipocytes permissive to SARS-CoV-2 infection ACE2 not detected in freshly isolated mature adipocytes ATM abortively infected (no production of infectious particles) Infection of SVC and adipocytes (VAT and SAT): ↑ ↑ ↑ IP-10, PDGFAA, PDGFAB/BB, I-4, IL-13 (SVC > adipocytes)
Saccon et al, 2022 (108)	Primary stromal-vascular cells from SAT or VAT differentiated into adipocytes	SARS-CoV-2 infects and replicates in mature adipocytes (VAT and SAT) Susceptibility to SARS-CoV-2 infection: VAT > SAT adipocytes (↑↑↑ production of infectious particles) ACE2 expression: VAT > SAT adipocytes Proinflammatory markers after SARS-CoV-2 infection: VAT > SAT HSL expression: ↓ in SAT
Zickler et al, 2022 (175)	Human mesenchymal stem cells differentiated into mature adipocytes Golden hamsters	↑ ACE2 expression on adipocyte differentiation SARS-CoV-2 replication only detected in lipid-laden adipocytes Viral replication ↓ by tetrahydrolipstatin (lipase inhibitor) and atorvastatin Lipogenic gene (*Fasn*, *Acly*, and *Acaca*) expression ↓ in AT of infected hamsters
Bogard et al, 2023 (176)	Golden hamsters (young adult [2 mo] and aged [22 mo])	7 dpi ↓ SAT and VAT masses (absolute and relative) ↓ mean adipocyte size in SAT and VAT ↓ expression of lipogenic genes (*Fasn* and *Scd1*) in SAT and VAT (young adults) ↑ expression of FAO gene *Acadvl* in VAT Cell infiltrates and CLSs in SAT, not VAT 22 dpi ↓ absolute and relative SAT and ↓ absolute VAT (aged) ↓ mean adipocyte size in SAT and VAT (aged > young adults) Cell infiltrates and CLSs in SAT, not VAT (aged > young adults)

Abbreviations: AT, adipose tissue; ATM, adipose tissue macrophages; CLS, crown-like structures; dpi, days postinfection; EAT, epicardial adipose tissue; FAO, fatty acid oxidation; HSL, hormone-sensitive lipase; hpi, hours postinfection; IP-10, interferon-γ–induced protein-10; IRF1, interferon regulatory factor 1; LD, lipid droplet; PDGFAA, platelet-derived growth factor AA; PDGFAB/BB, platelet-derived growth factor AA; SAT, subcutaneous adipose tissue; SVC, stromal vascular cells; VAT, visceral adipose tissue; XBP-1s, stress-responsive transcription factor X-box binding protein 1.

^*a*^Granulocyte activation, phagocytosis, adaptive immune response.

^*b*^Production of infectious particles.

In addition to the adiponectin-to-leptin ratio, other mechanisms may contribute to systemic insulin resistance in individuals with severe COVID-19. SARS-CoV-2 infection upregulates the RE1-silencing transcription factor (REST), which modulates the expression of myeloperoxidase (MPO), apelin, and myostatin (191). Specifically, REST upregulates MPO, which was shown to increase the expression of genes involved in gluconeogenesis in hepatocyte cell lines (*G6pc*) and adipocytes (*Pck1*) and to induce insulin resistance in adipocytes, preadipocytes, and myotubes. REST also downregulates the expression of apelin and myostatin, which have opposite effects to MPO (191). A downregulation of the insulin/IGF signaling pathway might also contribute to insulin resistance in adipose tissue and other organs that orchestrate glucose metabolism. In murine adipose tissue, SARS-CoV-2 upregulates the interferon regulatory factor 1 (IRF1), which acts as both a transcriptional activator and repressor to regulate interferon and cytokine responses (173). Enhanced expression of IRF1 is associated with an impairment of the insulin/IGF pathway in adipocytes (Fig. 1) (173).

In addition, the elevation of circulating FFA in people with COVID-19 (145, 192, 193) reflects excessive lipolysis, which is likely due to adipose tissue insulin resistance, but can also result from stress-induced hormones such as cortisol or adrenalin, similar to sepsis (194). High levels of circulating long-chain polyunsaturated fatty acids and low levels of long-chain acylcarnitines have been also reported in individuals with COVID-19, suggesting an increased activity of phospholipase A2 (PLA2) (145), which is required for replication of other coronaviruses (195). Increased PLA2 activity leads to greater production of omega-6 polyunsaturated fatty acid-derived bioactive metabolites such as eicosanoids and oxylipins, which may enhance both SARS-CoV-2 propagation and the inflammatory response (196-198). Increased expression of PLA2 and zinc-alpha2 glycoprotein (a potent promoter of β-adrenergic-driven lipolysis) in adipose tissue and reduced levels of circulating adiponectin have previously been reported during intensive care for subarachnoid hemorrhage (199). An increase in zinc-alpha2 glycoprotein is also a risk factor for severe COVID-19 (odds ratio, 1.37; 95% CI, 1.14-1.66) (200). Notably, administration of lipolysis inhibitors reduced viral replication in mature adipocytes (175) and significantly reduced mortality, alleviated lung pathology, and blunted virus replication in SARS-CoV-2-infected hamsters (201).

Finally, COVID-19 may have a detrimental impact on body composition. Clinically relevant weight loss during COVID-19 is common even in mild forms (202) and is followed by weight regain with increased abdominal adiposity (203). In a study that compared changes in body composition and insulin resistance (as assessed by homeostasis model assessment of insulin resistance [HOMA-IR]) in people with or without COVID-19, the former showed an increase in percentage fat mass despite a reduction in BMI, and significant increases in fasting plasma glucose, insulin, and HOMA-IR compared to pre-COVID-19 (204). These data suggest an association of COVID-19 and possibly other factors, such as inactivity, reduced food intake during illness, and increased food intake during recovery, with worsening body composition and glucose metabolism.

In summary, adipose tissue, particularly visceral adipose tissue, is a main target of SARS-CoV-2, which may induce adipose tissue insulin resistance and adipocyte dysfunction, with altered secretion of cytokines and adipokines contributing to systemic insulin resistance.

### Liver

#### Liver injury

Elevation of liver enzymes (alanine aminotransferase [ALT], aspartate aminotransferase [AST]) is a common finding in individuals with acute COVID-19, with up to 39% and 63% of individuals having mildly increased ALT or AST, respectively, and generally higher AST than ALT levels (205). The expression of known SARS-CoV-2 entry receptors and facilitators, including ACE2, TMPRSS2, procathepsin L, Ras-related protein Rab-7a, and GPR78 has been reported in liver autopsy samples from individuals who died from COVID-19 (206, 207). Because the ACE2 receptor is mainly expressed by cholangiocytes (208, 209) and Kupffer cells (207), the ability of SARS-CoV-2 to enter hepatocytes and exert cytopathic effects has been debated, also because of a paucity of histopathological studies having investigated the presence of the virus in liver samples (210). Some reported absence of viral RNA, proteins, or viral inclusions in hepatocytes (211-214), whereas others were able to detect viral inclusions or the SARS-CoV-2 spike protein (206, 207, 215, 216), viral entry possibly being facilitated by the high-density lipoprotein scavenger receptor class B member 1 (207, 217), a plasma membrane receptor for high-density lipoprotein cholesterol that also facilitates cell entry by the hepatitis C virus (218). Thus, liver injury is likely from both direct viral damage and other mechanisms, including immune dysregulation, inflammation, hypoxic/ischemic injury, and drug-induced hepatotoxicity (205).

#### Hepatic steatosis

In a systematic review and meta-analysis of autopsy studies, the most common histopathological finding was the presence of hepatic steatosis in 55% of liver samples from COVID-19-infected people (219). A more recent systematic review of histopathological studies reported that steatosis was detected in 42% of cases (210). These figures are greater than the prevalence of NAFLD in the general population (32.4%; 95% CI, 29.9-34.9) (220). A possible explanation is an over-representation of individuals with liver steatosis in autopsy series, owing to the association between NAFLD and severe COVID-19 (35). Nevertheless, this association disappears when demographic and comorbid factors are taken into account, with BMI and adiposity retaining a causal relationship with severe COVID-19 (221). Distinguishing between preexisting steatosis and SARS-CoV-2-induced steatosis is challenging.

**Figure 2.**
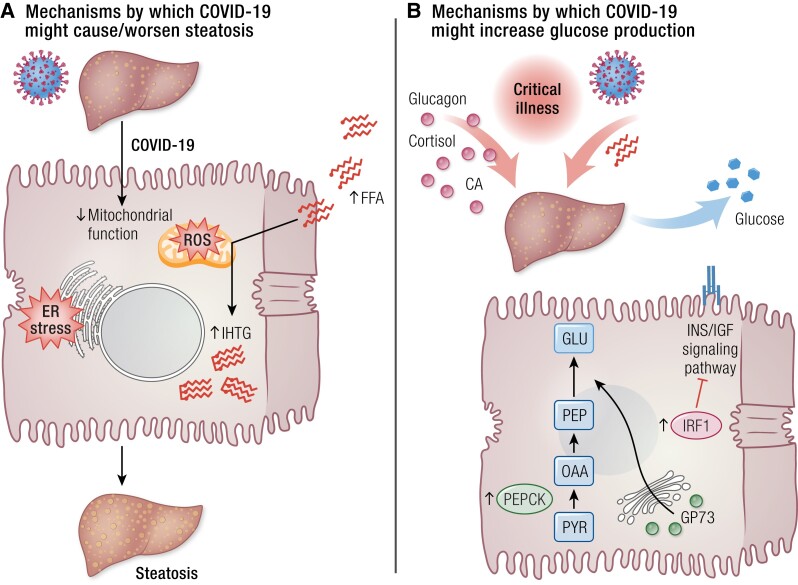
COVID-19 and the liver. (A) Mechanisms by which COVID-19 might cause or worsen hepatic steatosis. (B) Mechanisms by which COVID-19 might increase hepatic endogenous glucose production. Abbreviations: CA, catecholamines; ER, endoplasmic reticulum; FFA, free fatty acids; GLU, glucose; GP73, Golgi protein 73; IHTG, intrahepatic triglyceride; INS/IGF, insulin/insulin-like growth factor; IRF-1, interferon regulatory factor 1; OAA, oxaloacetate; PEP, phosphoenolpyruvate; PEPCK, phosphoenolpyruvate carboxykinase; PYR, pyruvate; ROS, reactive oxygen species.

Potential mechanisms by which COVID-19 might cause or worsen hepatic steatosis and other metabolic derangements are discussed in the following paragraphs and illustrated in Fig. 2. Hepatocellular ER stress is known to be involved in the pathophysiology of NAFLD (222, 223). In vitro, SARS-CoV-2 infection was shown to rapidly induce ER stress in human cell lines (224), and dilation of the ER in hepatocytes has been detected by transmission electron microscopy (211, 216, 225). Furthermore, the ER-resident chaperone GRP78, a stress marker involved in the unfolded protein response (a pathway preventing accumulation of unfolded and misfolded proteins by promoting autophagy and ER-associated degradation) that in ER stress conditions is translocated to the cell surface, where it can serve as a multifunctional receptor, including for virus entry (226, 227) mediates—at least in part—SARS-CoV-2 entry into hepatocytes (206).

Transmission electron microscopy of postmortem liver biopsies showed marked swelling of mitochondria in hepatocytes of individuals with COVID-19 (211, 216). Similarly to what has been observed in primary cells, cell lines, and biological samples from people with COVID-19 (228-231), SARS-CoV-2 infection in the liver induces downregulation of several genes responsible for oxidation reduction, oxidative phosphorylation, and cellular respiration (207, 232), indicating altered mitochondrial function. Hepatic mitochondria play a fundamental role in energy production via oxidation of amino acids, pyruvate, and fatty acids. Mitochondrial oxidative phosphorylation (ie, the coupling of substrate oxidation and ATP synthesis) is finely tuned and increases in response to substrate overflow (eg, in conditions of excess adiposity), a mechanism known as “mitochondrial plasticity” (233, 234). An impairment of this adaptation renders mitochondria unable to handle the excess flux of substrates to the liver and is involved in the development of NAFLD and its progression to nonalcoholic steatohepatitis and liver fibrosis (233). In fact, although people with obesity and NAFLD exhibit increased mitochondrial oxidative capacity compared with lean counterparts, individuals with nonalcoholic steatohepatitis (234) and those with type 2 diabetes or histological evidence of liver fibrosis (235) lose the ability to adapt their oxidative capacity to increased lipid loading. Fatty acid β-oxidation was found to be both upregulated (236, 237) and downregulated (232) in autoptic liver samples from individuals with COVID-19, which may reflect different stages of fatty liver disease (237, 238). Furthermore, higher levels of circulating FFA were found in individuals with critical COVID-19 compared with those with mild disease or no SARS-CoV-2 infection (145, 192), being associated with lipotoxicity and worse inflammation, intravascular thrombosis, organ failure, and mortality (192). Upregulation of fatty acid biosynthesis has also been detected in the liver of individuals with COVID-19 (236). Overall, the evidence supports ER stress and abnormal hepatic mitochondrial function in COVID-19. These mechanisms are established contributors to NAFLD (222, 223, 233) and favor hepatic lipid accumulation and excess oxidative stress. This mechanism has served to explain the development or worsening of fatty liver disease in people with COVID-19 (239). The results of a study that examined 235 individuals who had been hospitalized for severe COVID-19 and were reassessed approximately 5 months from symptom onset (240) are consistent with a steatosis-promoting effect of COVID-19. NAFLD was present in 55.3% (as diagnosed by transient elastography) to 71% (using the hepatic steatosis index) of participants at follow-up and in 37.3% (as calculated using proportional estimates between hepatic steatosis index and transient elastography data, *P* < .001) to 48% on admission.

#### Glucose metabolism

The liver plays a major role in glucose homeostasis as the main site for gluconeogenesis (82, 241). Excessive lipid availability induces insulin resistance in the liver, which manifests as the inability of insulin to suppress hepatic gluconeogenesis and glucose release in the circulation, thus contributing to fasting hyperglycemia and eventually type 2 diabetes (242). During critical illness, cytokines and other pathways give rise to release of catabolic hormones such as glucagon, catecholamines, and cortisol, which stimulate glycogenolysis and/or gluconeogenesis to provide glucose for tissues and cells that use it as their primary energy source (243) (Fig. 2). An increase in circulating FFA such as reported in COVID-19 (145, 192, 193, 244), enhances fatty acid delivery to the liver, which in turn stimulates gluconeogenesis via allosteric regulation of key enzymes or substrate (glycerol) availability (82). Other mechanisms, more specific to SARS-CoV-2 infection, are reported to contribute to increased gluconeogenesis and subsequent hyperglycemia during COVID-19 (Fig. 2). As mentioned, acute infection with SARS-CoV-2 upregulates the expression of IRF1, leading to downregulation of the insulin/IGF signaling pathway genes in human pluripotent stem cell-derived human liver organoids, mouse adipose tissue, and induced-pluripotent stem-derived human pancreatic cells (173). Consistent with these in vitro observations, the gene expression of IRF1 was significantly increased and that of insulin/IGF signaling pathway was downregulated in the whole blood of individuals with critical COVID-19 (173). Recently, 2 novel mechanisms underlying excess endogenous glucose production have been described. First, SARS-CoV-2 infection stimulates glucose production by increasing the activity of phosphoenolpyruvate carboxykinase in primary human hepatocytes (206). The second mechanism involves the Golgi protein-73 (GP73), a type II transmembrane protein located at the luminal surface of the Golgi apparatus that is released in the circulation and regulates intercellular communication during the unfolded protein response (245). Both fasting and a high-fat diet were shown to increase levels of circulating GP73 in mice, which stimulated hepatic gluconeogenesis (246). Similar to fluctuations in nutrient status, SARS-CoV-2 infection promoted GP73 production and secretion in cultured hepatocytes and in mice, inducing hepatocyte glucose production and hyperglycemia in mice, whereas GP73 blockade inhibited SARS-CoV-2-induced increases in gluconeogenesis in vitro and lowered elevated fasting blood glucose levels in infected mice (246). Elevated circulating levels of GP73 were found in people with COVID-19, the levels being higher in those with more severe disease and positively correlating with glucose levels (246). Levels of GP73 and glucose remained elevated on hospital discharge, which could indicate that GP73 is implicated in the persistence of hyperglycemia after recovery from COVID-19.

#### Ketogenesis

Individuals with COVID-19 also feature increased levels of ketone bodies in serum and urine (247-250). Ketogenesis (ie, the production of ketone bodies from acetyl coenzyme A derived from β oxidation of FFA) occurs in hepatic mitochondria when glucose availability is low, providing an energy substrate alternative to glucose. The liver is the only organ that can provide enough ketone bodies to support survival under ketogenic conditions (251). Plasma β-hydroxybutyrate was found to be elevated on hospital admission in individuals with COVID-19, increased with worsening disease, but decreased within a week during improved clinical disease (252) and on recovery from COVID-19 (248). Increased ketogenesis is not specific for SARS-CoV-2 infection because any critical illness induces catabolism with greater availability of FFA (253). Of note, the ketone body β-hydroxybutyrate was also shown to exert anti-inflammatory and antioxidant effects (254). In this light, increased ketogenesis might be an adaptative mechanism to dampen inflammation and oxidative stress. In mice infected with murine beta coronavirus A59, a ketogenic diet resulting in mild physiological ketosis prevented infection-induced weight loss and hypoxemia, improved survival, and reduced the expression of the NLRP3 inflammasome and pro-inflammatory cytokines (IL-1β, TNFα, IL-6) (126). In line with this, a previous study showed that a high-fat ketogenic diet protects mice against influenza by enhancing antiviral resistance through expansion of protective γδ T cells (255). Preclinical studies also indicate that β-hydroxybutyrate inhibits activation of the NLRP3 inflammasome in isolated hearts and in the macrophages of murine models of systemic inflammatory diseases or Alzheimer disease (256-259). Nevertheless, studies in humans yielded conflicting results (260-262). It has been reported that β-hydroxybutyrate enhances the antiviral innate immune response by promoting the secretion of IFN-γ from T cells obtained from individuals with COVID-19 (263, 264). Levels of β-hydroxybutyrate are higher in individuals with moderate COVID-19 or COVID-19-related ARDS than in healthy individuals, but significantly lower than in individuals with influenza-related ARDS. These differences were independent of glucose or insulin levels and suggest a blunted ketogenic response to SARS-CoV-2 infection (264).

The observed increase in ketogenesis in individuals with COVID-19 (247-250, 252) is consistent with an increase in gluconeogenesis. Oxaloacetate, which is necessary for β-oxidation-derived acetyl-CoA to form citrate and enter the tricarboxylic acid cycle, is used as a precursor of glucose in gluconeogenesis. When availability of oxaloacetate is low, β-oxidation-derived acetyl-CoA cannot enter the tricarboxylic acid cycle and is rerouted to the ketogenic pathway (254). Of note, plasma glucose levels were reported to parallel changes in ketone body levels, with persistent hyperglycemia in people with more severe disease, and normalization of glucose levels in those with favorable outcomes (252).

In summary, acute COVID-19 associates with multiple metabolic alterations in the liver, spanning from excessive lipid influx and mitochondrial damage to ER stress causing lipid-accumulation and enhanced gluconeogenesis with increased endogenous glucose production.

### Skeletal Muscle

**Figure 3.**
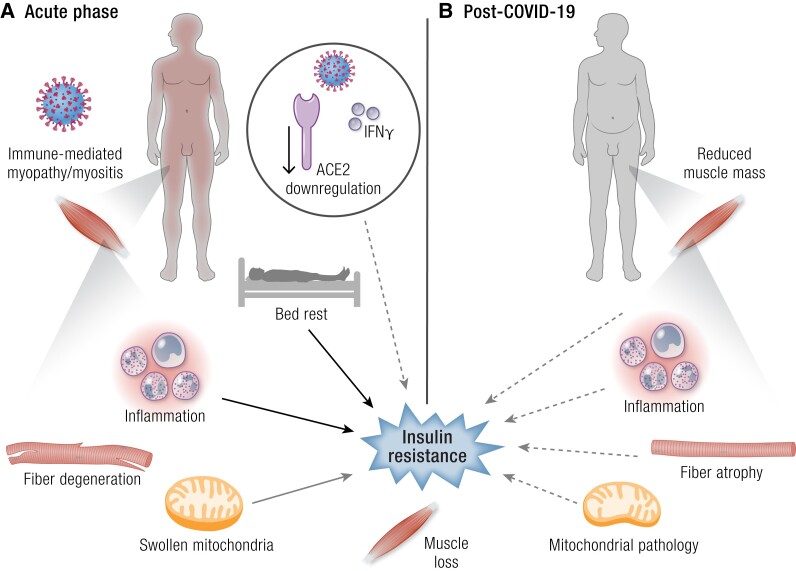
COVID-19 and the skeletal muscle. (A) Effects of acute COVID-19 on skeletal muscle. (B) Potential consequences of COVID-19 on skeletal muscle. Dashed arrows indicate hypothesized mechanisms. Abbreviations: ACE, angiotensin-converting enzyme 2; ADN, adiponectin; LEP, leptin.

Observational studies indicate that muscle damage is common during acute COVID-19, as indicated by elevated creatine kinase levels in 17% of infected people (265). Muscular symptoms such as myalgia (muscle pain) and muscle weakness are also common, both in acute COVID-19 and in PASC (266). Evidence indicates that, in the acute phase, skeletal muscle damage is due to an immune-mediated myopathy/myositis rather than a direct cytopathic effect of SARS-CoV-2 (267, 268) (Fig. 3). A case-control autopsy series demonstrated that skeletal muscle from individuals who had died with COVID-19 had more signs of muscle inflammation and degenerating muscle fibers than people who had died from other causes (267). Individuals with COVID-19 also had significantly greater infiltration of CD45-positive leukocytes and CD8-positive T cells, but no evidence of a direct viral infection of myofibers, which is consistent with the findings of another histopathology study (268). Swollen mitochondria and elevation of circulating growth differentiation factor 15 (269), which is a marker of skeletal muscle bioenergetic dysfunction released from muscle in response to mitochondrial proteotoxic stress and activation of the mitochondrial unfolded protein response (270-272), provide evidence of mitochondrial damage in the skeletal muscle of individuals with critical COVID-19. Fatigue or muscle weakness are among the most common long-term symptoms following COVID-19 (273). In muscle biopsies from individuals with persistent muscle symptoms after up to 14 months since recovery from COVID-19, degenerative alterations including myofiber atrophy were common (274). Signs of inflammation and mitochondrial pathology such as loss of cytochrome C oxidase activity, subsarcolemmal accumulation, and/or abnormal cristae, were also present in some biopsies (Fig. 3).

Insulin resistance has been reported in COVID-19, both during the acute phase and after recovery (Table 3). Skeletal muscle is the main site of insulin-stimulated glucose disposal; therefore, muscle insulin resistance has a predominant impact on whole-body glucose metabolism. Alterations in skeletal muscle mass and physiology play an important role in the insulin resistance leading to dysglycemia during critical illness (107). Acute systemic inflammation during infections profoundly impairs insulin-stimulated whole-body glucose disposal, endogenous glucose production, and glucose oxidation (281). Alterations in cytokine and adipokine profiles such as those described in people with severe COVID-19 (107, 178, 179) are known to affect insulin signaling pathways and GLUT4 translocation and to impair insulin-mediated glucose uptake in skeletal muscle (282).

**Table 3.** Studies that assessed indexes of glucose metabolism (insulin sensitivity/resistance and/or β-cell function) in adults with COVID-19

Author	Design	Timing	Outcome measure(s)	Main findings
Alshammari et al 2023 (275)	D: longitudinal P: COVID-19 (n = 65) DM: yes (100%) C: N/A M: 46.2% S: N/A	Before, during, and after complete remission of COVID-19 (timing not specified)	HbA1c TyG TyG-BMI TG/HDL METS-IR Comparisons: before, during, and after COVID-19	HbA1c: 7.9 ± 0.2 vs 10.1 ± 0.4 vs 8.4 ± 0.3%, TyG: 9.5 ± 0.04 vs 10.0 ± 0.05 vs 9.7 ± 0.05, TyG-BMI: 312.7 ± 7.7 vs 326.8 ± 8.0 vs 318.1 ± 7.9 TG/HDL: 3.9 ± 0.1 vs 4.8 ± 0.1 vs 4.4 ± 0.1 METS-IR: 54.9 ± 1.3 vs 58.0 ± 1.4 vs 55.9 ± 1.3
Barreto et al 2023 (206)	D: cross-sectional P: COVID-19, hospitalized (n = 647) DM: no C: COVID-19 negative, hospitalized (n = 203) M: 55.3% S: moderate (14.2%) or severe (85.8%)	Acute phase	C peptide Glucagon Comparisons: COVID-19 neg. (n = 17) vs normoglycemic COVID-19 positive (n = 39) vs COVID-19 positive with high BG (n = 63) All without DM	> C peptide and glucagon in COVID-19 with hyperglycemia vs non-COVID-19 > C peptide in COVID-19 with hyperglycemia vs COVID-19 with normoglycemia
Chen 2021 et al (276)	D: longitudinal P: COVID-19, hospitalized (n = 64) DM: no C: N/A M: 54.7% S: 84.4% mild/moderate, 15.6% severe/critical	Acute phase, 3 and 6 mo. after	C peptide (fasting) HOMA-CP TyG HOMA-IR FBG	C-peptide: ↑ (0.35 ± 0.24 vs 2.36 ± 0.98 vs 2.52 ± 1.11 μg/L) HOMA-CP: ↑ (median [IQR] 0.42 [0.36-0.62] vs 2.54 [1.95-3.42] vs 2.90 [2.02-4.23]) TyG: ↑ (8.57 ± 0.47 vs 8.73 ± 0.60 vs 8.82 ± 0.62) HOMA-IR: ⇌ FBG: ↓ (105.1 ± 21.8 vs 89.1 ± 13.7 vs 97.2 ± 12.2 mg/dL)
Gojda et al 2023 (193)	D: longitudinal P: COVID-19, hospitalized DM: N/A C: N/A M: 65% S: Severe	Acute phase, 3 and 6 mo after	C peptide II ISI DI HOMA-B HOMA-IR Comparisons: baseline vs 6 mo. normoglycemic vs hyperglycemic	Baseline vs 6 mo (all) C peptide, DI: ⇌ ISI: 3.23 ± 1.41 vs 4.53 ± 2.30 HOMA-B: 1.59 [1.14-2.50] vs 1.01[0.81-1.53] Normoglycemic vs hyperglycemic (baseline) II: 2.78 ± 1.77 vs 1.01 ± 0.51 DI: 8.40 ± 5.42 vs 3.05 ± 1.79 ISI, HOMA-B, HOMA-IR: = Normoglycemic vs hyperglycemic (6 mo.) II, ISI, SI, HOMA-B, HOMA-IR: =
Goyal et al 2021 (277)	D: longitudinal P: SARS-CoV-2 infection (n = 159) DM: no C: noninfected people (n = 193) M: 46.9% S: asymptomatic/mild (76.7%)	Pre-COVID period (years 2016-2019) and peri-COVID period (years 2020-2021). Mean FU 21.5 ± 7.1 mo (regardless of the timing of COVID-19 diagnosis)	HOMA-IR Matsuda index DI Comparisons: changes from baseline to FU in noninfected vs infected	HOMA-IR: ⇌ Matsuda index: ⇌ DI: ⇌
He et al 2021 (191)	D: longitudinal P: COVID-19, hospitalized (n = 92) DM: no C: HC (n = 30) M: 41.3% S: 87.0% nonsevere, 13% severe	Acute and recovery phase (timing not specified)	FBG Insulin (fasting) HOMA-IR HDL-C	> FBG, insulin and HOMA-IR, and < HDL-C in people with COVID-19 vs HC during the acute phase (values not reported) > FBG and < HDL-C in people with COVID-19 vs HC during the recovery phase (values not reported)
Ilias et al 2021 (278)	D: cross-sectional P: COVID-19, hospitalized (n = 157) DM: yes (14.0%) C: N/A M: 71.3% S: 53.5% nonsevere (ward), 46.5% severe (ICU)	Acute phase	aBG HbA1c Insulin (admission) C peptide (admission) HOMA2%B HOMA2%S Comparisons: ICU (+/− DM) vs ward (+/no DM)	aBG: lowest in ward-DM vs ward + DM vs ICU-DM vs ICU-DM (median [IQR] 101 [84-116] vs 136 [113-171] vs138 [110-184] vs 192 [149-275] mg/dL) HOMA2%B > in ward −/+ DM vs ICU −/+ DM (103.2 ± 63.2 and 89.0 ± 84.5 vs 76.9 ± 63.2 and 44.0 ± 19.0%) HbA1c, insulin, C peptide, HOMA2%S: =
Milic et al 2022 (240)	D: cross-sectional P: COVID-19, hospitalized (n = 235) DM: yes (14.0%) C: N/A M: 69% S: 19.2% severe (IMV or NIV)	143 (median) [IQR, 130-163.5] days after symptom onset	HOMA-IR	HOMA-IR (median [IQR]): 2.2 [1.4-3.8]
Montefusco et al 2021 (279)	D: retrospective, longitudinal P: COVID-19, hospitalized (n = 20) DM: no C: HC (n = 15) M: 62% S: N/A	Acute phase and 62.0 ± 6.5 days after disease onset	CGM Insulin (fasting) C peptide HOMA-B HOMA-IR Arginine stimulation test	Acute COVID vs HC CGM: longer BG > 140 mg/dL, > BG AUC >140 mg/dL, > mean postprandial BG at 60 minutes, > glycemic variability Insulin (fasting): > vs HC C peptide: > vs HC HOMA-B: > vs HC HOMA-IR: > vs HC Arginine test: > AIRmax, > insulin and C peptide_AUC_ Post-COVID-19 vs HC CGM: longer duration of BG > 140 mg/dL, > mean postprandial BG at 120 min, > mean blood glucose, > nadir BG Insulin (fasting): > vs HC C peptide: > vs HC HOMA-B: > vs HC HOMA-IR: > vs HC Arginine test: > AIRmax, > insulin_AUC_
Reiterer et al 2021 (107)	D: retrospective, cross-sectional P: COVID-19, hospitalized (n = 59) DM: 28.8% C: ICU, no COVID-19 (n = 42, 42.9% with ARDS) M: 78% S: severe (ARDS)	Acute phase	Insulin (fasting) C peptide C-peptide/glucose ratio Insulin resistance (based on C-peptide/glucose ratio) β cell failure (based on C-peptide/glucose ratio) Comparisons: COVID-19 vs ICU + ARDS vs ICUnoARDS	Insulin (fasting, median [IQR]): = C peptide (median [IQR]): highest in COVID-19: 3487 [1552-5756] vs 1436 [851-2819] vs 1437 [916-2014] pg/mL C-peptide/glucose ratio highest in COVID-19 Insulin resistance: 57.6% vs 16.7% vs 8.3% β-cell failure: 35.6% vs 55.6% vs 25.0%
Soto et al 2022 (280)	D: cross-sectional P: COVID-19, hospitalized (n = 61) DM: yes (26%) C: HC (n = 25) M: 72% S: moderate (55.7%) or severe (44.3%)	Acute phase	Insulin (fasting) FBG HOMA-IR	> insulin, FBG, HOMA-IR in COVID-19 vs HC
Yazdanpanah et al 2023 (204)	D: longitudinal P: COVID-19, nonhospitalized (n = 221) DM: C: non-COVID-19 (n = 220) M: 56.1% S: mild to moderate (no hospitalization)	Before and after COVID-19 (one month since the baseline assessment, regardless of the timing of COVID-19 diagnosis)	HOMA-IR Comparisons: non-COVID-19, pre-post COVID-19, pre-post	COVID-19 HOMA-IR: 0.92 ± 0.3 vs 1.23 ± 0.28 Non-COVID-19 HOMA-IR: 0.91 ± 0.3 vs 0.8 ± 0.21

Legend: ↑ increase; ↓ decrease; ⇌ no change; > higher; < lower; = no difference.

Abbreviations: aBG, blood glucose on admission; AIRmax, acute insulin responses to arginine; ARDS, acute respiratory distress syndrome; AUC, area under the curve; C, controls; CGM, continuous glucose monitoring; D, design; DI, disposition index; DM, diabetes mellitus; FBG, fasting blood glucose; FU, follow-up; HC, healthy controls; HDL-C, high-density lipoprotein cholesterol; HOMA, homeostatic model assessment; HOMA2%B, homeostatic model assessment 2 for β-cell function; HOMA2%S, homeostatic model assessment 2 for insulin sensitivity; HOMA-CP, homeostatic model assessment for β-cell function (using C peptide); HOMA-IR, homeostatic model assessment for insulin resistance; ICU, intensive care unit; II, insulinogenic index; IMV, invasive mechanical ventilation; IQR, interquartile range; ISI, insulin sensitivity index; M, males (%); METS-IR, metabolic score for insulin resistance; NIV, noninvasive mechanical ventilation; N/A, not available; P, participants; S, severity; TG/HDL, triglyceride to high-density lipoprotein cholesterol ratio; TyG, triglyceride-glucose index; TyG-BMI, triglyceride glucose-body mass index.

Muscle wasting resulting from muscle catabolism for the provision of amino acids as substrates for tissue repair and synthesis of proteins for the immune response is a detrimental consequence of acute inflammation (283). Bed rest also causes muscle loss, mainly because of suppression of muscle protein synthesis (284, 285). Acute loss of muscle mass during COVID-19 requiring hospitalization is even greater than in other catabolic conditions and impacts short- and long-term clinical outcomes (286, 287). A substantial loss of muscle mass secondary to muscle disuse results in reduced whole-body glucose utilization (288, 289), possibly contributing to hyperglycemia both in acute COVID-19 and after recovery. Acute (3-day) bed rest results in a 17% reduction in insulin-stimulated whole-body glucose disposal, which worsens only slightly when bed rest is prolonged further (up to 60 days) in healthy, normal weight men (289). The decrease in glucose disposal is paralleled by inhibition of muscle glycogen storage, and by blunted carbohydrate oxidation with longer bed rest, but not by an increase in intramyocellular lipid content. To the best of our knowledge, no studies have specifically investigated the relationship between muscle mass loss and glucose metabolism in COVID-19. In a study examining the prevalence of reduced muscle mass among COVID-19 survivors, the proportion of individuals with diabetes was significantly higher in those with compared with those without reduced muscle mass (16% vs 6%, respectively; *P* = .001), and having diabetes was associated with increased odds of reduced muscle mass (290). These data suggest an association between a reduction in skeletal muscle mass and impaired glucose metabolism (291). Of note, significant loss of muscle mass and strength (ie, sarcopenia) during hospitalization associates with failure to return to baseline values at 6 months after discharge, and increased likelihood of PASC symptoms such as fatigue and myalgia (287).

COVID-19-specific mechanisms such as increased levels of IFN-γ (292) and reduction of ACE2 levels (293, 294) following SARS-CoV-2 infection could contribute to insulin resistance in skeletal muscle, although specific evidence is lacking. Virus-induced IFN-γ leads to downregulation of the insulin receptor in skeletal muscle, thus affecting insulin sensitivity (295). In rodent models, blockade of ACE2 decreases insulin-stimulated glucose uptake and microvascular blood flow in muscle and insulin receptor signaling (PI3K/Akt activation) in the liver and adipose tissue (296, 297), and exacerbates diet-induced glucose intolerance and insulin resistance by reducing the expression of GLUT4 in skeletal muscle (298). Angiotensin 1-7, which is generated from angiotensin II by ACE2, has protective effects on skeletal muscle, mainly because of antagonism of the pro-atrophic and pro-fibrotic actions of angiotensin II on muscle (299). Downregulation of ACE2 in COVID-19 leads to decreased angiotensin 1-7 and increased angiotensin II levels (300), possibly worsening skeletal muscle damage and loss associated with inflammation and immobilization.

In summary, skeletal muscle damage in COVID-19 appears to be mainly due to immune-inflammatory mechanisms. Mitochondrial damage may contribute to impaired muscle bioenergetics (269) and to the persistence of common PASC symptoms such as fatigue and myalgia (274). Both critical illness and SARS-CoV-2-related mechanisms such as an increase in IFN-γ and ACE2 downregulation impair insulin sensitivity in skeletal muscle during COVID-19. Less information is available on the post-recovery phase. It can be hypothesized that a reduction in muscle mass from acute systemic inflammation, disuse, and possibly malnutrition contributes to persistence of impaired whole-body glucose disposal in some people.

### Pancreas

**Figure 4.**
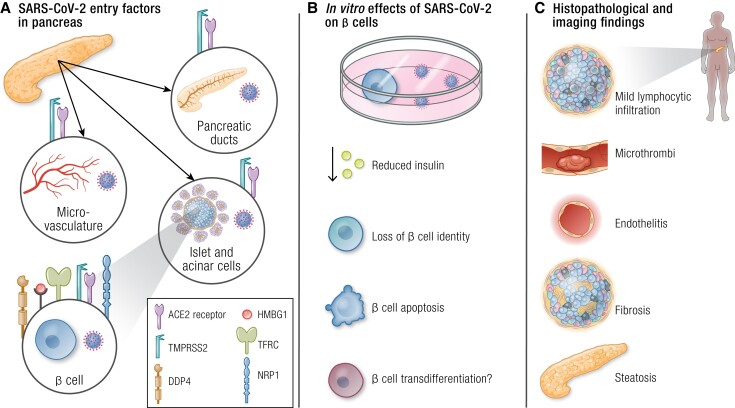
COVID-19 and the pancreas. (A) SARS-CoV-2 entry factors in pancreatic cells; (B) in vitro effects of SARS-CoV-2 on β cells. (C) Histopathological and imaging findings in people with COVID-19. Abbreviations: ACE2, angiotensin-converting enzyme 2; DPP4, dipeptidyl-peptidase 4; HMBG1, high-mobility group box 1 protein; NRP1, neuropilin 1; TFRC, transferrin receptor; TMPRSS2, transmembrane protease serine 2.

Evidence of pancreatic injury (increases in amylase and lipase, and focal enlargement of the pancreas or dilation of the pancreatic duct on computed tomography) in people with COVID-19 was reported soon after the beginning of the pandemic (301).The pancreas has been therefore identified as one of the target organs of SARS-CoV-2 (Fig. 4). SARS-CoV-2 viral antigen has been identified in pancreatic islets of autopsy samples from individuals with COVID-19 (302, 303), indicating that the virus can directly infect the pancreas. Immunohistochemistry and immunofluorescence of pancreata from persons who died from COVID-19 suggest that SARS-CoV-2 infection of islets induces necroptosis (302), a form of regulated necrosis mediated by receptor interacting protein kinase-3 and its substrate mixed lineage kinase like (304), and infiltration of immune cells with local inflammation (302, 305). Localization of ACE2 in pancreatic islets was demonstrated more than a decade ago, when investigating the pathophysiology of SARS-CoV-associated diabetes (44). Most (301-303, 306-309)—although not all (310, 311)—studies found evidence of ACE2 or TMPRSS2 expression on pancreatic endocrine cells. Nonetheless, greater expression of ACE2 has been reported in pancreatic microvascular and ductal structures (302, 311). It should be noted, however, that SARS-CoV-2 antigens were detected in endothelial, exocrine, and endocrine cells (β and non-β cells), independent of ACE2 expression (302). Dipeptidyl-peptidase 4, high-mobility group box 1 protein, transferrin receptor, and NRP1 have been identified as alternative SARS-CoV-2 entry factors, and are expressed in pancreatic islets (302, 303, 312-315) (Fig. 4).

In vitro, SARS-CoV-2 decreases insulin content and glucose-stimulated insulin secretion of infected islets (303, 307), induces loss of β-cell identity (307) and β-cell apoptosis via the c-Jun N-terminal protein kinase MAPK pathway (303) (Fig. 4). In vitro studies indicate that the inflammatory milieu characteristic of COVID-19 contributes to β-cell damage. Exposure of cultured human pancreatic islets to serum obtained from humans hospitalized for COVID-19 with new-onset hyperglycemia or who had recovered from COVID-19 led to islet apoptosis and a dramatic reduction in insulin secretion, which were mediated by proinflammatory cytokines including IL-1β, IL-6, IL-13, IP-10, and TNF-α (305). It has also been proposed that transdifferentiation of β cells following SARS-CoV-2 infection leads to lower insulin and greater glucagon production (313). However, a subsequent study failed to confirm that SARS-CoV-2 infection of β cells causes transdifferentiation and indicated only limited, noncytopathic infection, a modest inflammatory response, and minor cellular perturbations, questioning the diabetogenicity of SARS-CoV-2 (309). Overall, most preclinical studies indicate that SARS-CoV-2 has the potential to impair insulin secretion via both direct and indirect β-cell damage. Although the evidence from autopsy studies indicates that the pancreas can be infected by SARS-CoV-2, the amount of viral RNA in the pancreas is lower than in other tissues (302, 316), the direct endocrine cell damage, if any, is mild (302, 305), viral persistence in the organ is short (316), and no histopathology data are available for COVID-19 survivors.

Very few clinical studies have investigated the pathophysiological mechanisms of hyperglycemia in COVID-19 (Table 3). In a study including ∼5500 individuals admitted to an intensive care unit, of whom ∼800 had COVID-19 and ∼4700 were SARS-CoV-2 negative, with or without ARDS (107), the prevalence of hyperglycemia was similarly high among persons with ARDS, whether they had COVID-19 (86%) or not (86%) (107). People with COVID-19 had significantly higher levels of plasma C-peptide than controls. Elevated C-peptide and hyperglycemia indicate that β cell function was preserved and insulin resistance is the predominant mechanism underlying hyperglycemia in COVID-19. A more recent but smaller study reinforces these findings: individuals with COVID-19 had hyperglycemia, hyperinsulinemia, and insulin resistance (as assessed by HOMA-IR), which was related to an increase in oxidative and nitrosative stress (280). Similarly, higher mean fasting insulin, proinsulin, C peptide levels, homeostasis model assessment of β-cell function, and HOMA-IR have been found in individuals with COVID-19 compared with healthy controls (279). These differences persisted after recovery from COVID-19. The findings of another study (276) showing that C peptide levels were below the lower limit of normal on admission, are somehow in contrast with reports of hyperinsulinemia during COVID-19. Differences could be attributed to different timing of measurements and different disease severity. Mean C peptide levels returned to normal values at 3 months after discharge and thereafter (from 0.35 at baseline to 2.36 and 2.52 at 3 and 6 months, respectively), whereas insulin resistance persisted and even increased up to 6 months (Table 3) despite reduced systemic inflammation, as assessed by C-reactive protein (276). A recent study found that, in individuals with severe COVID-19, inadequate insulin secretion in the face of increased insulin resistance (lower disposition index) was responsible for hyperglycemia during the acute phase (193). At 6 months after acute COVID-19, insulin resistance and β-cell function had both improved, and indexes of glucose metabolism were comparable between individuals with or without hyperglycemia during the acute phase. The response to an oral glucose challenge, however, revealed greater glucose values in the hyperglycemic group. Finally, the results of a small, retrospective study in people with type 2 diabetes who had available data before, during, and after COVID-19, suggest that insulin resistance is increased during the acute phase of disease and decreases after recovery from COVID-19, but remains higher compared with baseline (275). Despite some limitations (sample size, use of surrogate indices that may be influenced by medications, such as the triglyceride-glucose index, lack of information on the time of follow-up), this study is probably the only one to date that has compared glycemic control in individuals with type 2 diabetes before, during, and after COVID-19. Its results build on previous research (279), suggesting that perturbations in glucose metabolism persist even after remission. Of note, SARS-CoV-induced β-cell damage was shown to persist up to 3 years (44), and alterations in glucose metabolism have been detected in more than half of individuals in a small study with a follow-up of up to 12 years (43) after recovery from acute SARS-CoV infection. Importantly, studies that estimated insulin sensitivity/resistance and/or β-cell function (Table 3) have several limitations. Most included a relatively low number of participants, time of blood sampling since hospital admission and definition of hyperglycemia were not standardized, some did not adjust for between-group differences in age, BMI, preexisting diabetes, or other important baseline characteristics (107, 191, 278) did not provide a description of the non-COVID-19 control group (280), did not specify the time of follow-up (191, 275), did not make a statistical comparison of longitudinal differences in insulin resistance between people with and without COVID-19 (204), did not take the effects of steroid and/or insulin therapy during hospital stay into account (191, 276, 278), did not assess/adjust for longitudinal changes in BMI and/or body composition, performed pre-post comparisons using data from different individuals (279), had highly variable follow-up since SARS-CoV-2 infection/COVID-19 (204, 277), or used nonstandardized definitions to classify participants as insulin resistant (107).

It should also be considered that pancreatitis, which is a cause of pancreatogenic (type 3c) diabetes (317), is relatively common during COVID-19, with approximately 25% of individuals showing elevated pancreatic enzymes (318). Evidence of endothelitis, microthrombi, and fibrosis in pancreata from people who died from COVID-19 has been reported (319). Nevertheless, the majority of autopsy studies found a low rate of focal pancreatitis or islet degeneration (320-322). In people who survived COVID-19 and had persistent symptoms, a magnetic resonance imaging study found that nearly 40% showed fat accumulation and 15% showed signs of inflammation in the pancreas after a median of 141 days since initial symptom onset (323). These rates were significantly greater than in non-COVID-19 controls and in people hospitalized for COVID-19 vs those who were not hospitalized. Although the authors did not investigate the relationship between pancreatic impairment and metabolic abnormalities, the proportion of individuals with diabetes in the whole cohort was relatively low (2%). Whether intrapancreatic ectopic fat is associated with reduced insulin secretion and increased risk of type 2 diabetes is still debated, with some (324, 325) but not all studies suggesting an association between pancreas steatosis and β-cell function (326, 327). Further studies should investigate whether pancreas steatosis negatively impacts glucose metabolism in COVID-19 survivors.

In summary, although most in vitro studies suggest that SARS-CoV-2 might profoundly impact β-cell function and survival, evidence from autopsy studies indicates that pancreatic damage is infrequent and mild (302, 305, 310, 320-322), and a cause-and-effect relationship between histopathological/imaging findings and disruption of glucose metabolism has yet to be proven. Most of the available clinical studies (107, 191, 206, 275, 276, 278-280) indicate that insulin resistance rather than β-cell failure is the main driver of hyperglycemia in COVID-19. During the acute phase, inadequate insulin secretion in the presence of insulin resistance (193, 276, 278) likely contributes to the development of hyperglycemia. This could contribute to diabetic ketoacidosis, which develops when there is an absolute or relative insulin deficiency and—although its incidence is low—is associated with increased mortality in COVID-19 (328). Notably, during diabetic ketoacidosis, insulin resistance was greater in pediatric patients with COVID-19 and preexisting type 1 diabetes compared with those without COVID-19 (329). Establishing whether hyperglycemia during COVID-19 is due, at least in part, to SARS-CoV-2-specific mechanisms or rather to the inflammatory burden during acute illness (ie, stress hyperglycemia) is complex. A study of patients admitted to the intensive care unit with SARS demonstrated that those with COVID-19 had greater alterations in glycemic parameters than those with SARS from other causes (330). However, after adjustment for multiple confounders, glycemic alterations were associated with indicators of disease severity rather than with COVID-19. Stress-induced hyperglycemia during hospital stay is an independent risk factor for post-discharge incident diabetes (331-333). Similar to reports on COVID-19 (59), among individuals with newly diagnosed diabetes after stress-induced hyperglycemia the proportion of those with persistent hyperglycemia decreases over time, although as high as two thirds remain hyperglycemic (334). Both impaired β-cell secretory capacity and reduced insulin sensitivity have been implicated in the persistence of impaired glucose metabolism following stress hyperglycemia (334). Pre-existing functional alterations of β cells such as impaired β-cell glucose sensitivity and early-phase insulin secretion could be responsible for the impairment of glucose metabolism rather than β-mass reduction (335) because of SARS-CoV-2 toxicity. That is, in the presence of an acute event—such as critical illness—people with preexisting, subclinical impairment of β-cell function cannot cope with the sudden increase in insulin demand and develop hyperglycemia. Consistent with preexisting β-cell dysfunction, people with severe COVID-19 who develop hyperglycemia exhibit a worse glycemic response because of a lower insulinogenic response, dominantly in the early phase (30 minutes) of an oral glucose challenge (193). Studies in mice indicate that insulin resistance in skeletal muscle is compensated by hyperinsulinemia when glucose metabolism is normal, but prediabetic mice with insulin resistance caused by diet-induced obesity develop hyperglycemia because of insufficient compensation (295). Consistently, the risk of being diagnosed with type 2 diabetes increases with increasing pre-COVID-19 HbA1c levels (HR, 4.08 [95% CI, 2.81-5.34], *P* < .001) (336).

### Corticosteroid Therapy

Early administration of dexamethasone improves outcomes in patients with moderate-to-severe non-COVID-19 ARDS (337) and in those with COVID-19 requiring respiratory support (338). However, steroid-induced hyperglycemia is a common downside of steroid treatment in hospitalized individuals (339-341) that needs to be taken into account. Glucocorticoids have several detrimental effects on glucose metabolism, including reduced insulin-stimulated activity of the insulin receptor substrate 1-associated phosphoinositide 3-kinase, phosphorylation of insulin-signaling proteins, translocation of the glucose transporter type 4, insulin-stimulated glucose uptake, insulin-stimulated glycogen synthase, and increased glucose production (342, 343). Steroid use is a predictor of in-hospital (51) and persistent diabetes at 3 months (21) but not 5 months (51) after COVID-19 diagnosis. In analyses that excluded people who received steroid treatment, the risk of new-onset type 2 diabetes within 6 months of COVID-19 or influenza was still significantly increased in COVID-19 vs influenza, with steroid treatment apparently contributing to diabetes risk only in people with mild disease (Table 2) (53). Importantly, in people with less severe COVID-19 (ie, receiving no oxygen or simple oxygen), higher doses of corticosteroids were shown to have detrimental consequences and should be avoided (344). The benefits of steroid therapy in people with severe COVID-19 have been clearly demonstrated (338), and the fear of steroid-induced hyperglycemia should not hold one back from using it. Previous studies demonstrated that patients with community-acquired pneumonia treated with steroids had higher mean glucose levels and higher blood glucose variability vs patients treated with placebo, but these did not blunt the benefits of steroid therapy in hyperglycemic patients (345, 346). Similar findings have been reported in people hospitalized for COVID-19 (347). Furthermore, specific guidelines have been developed for the management of people with severe COVID-19 who are started on dexamethasone to ensure proper glucose monitoring and treatment of hyperglycemia (348).

## Connecting the Dots

**Figure 5.**
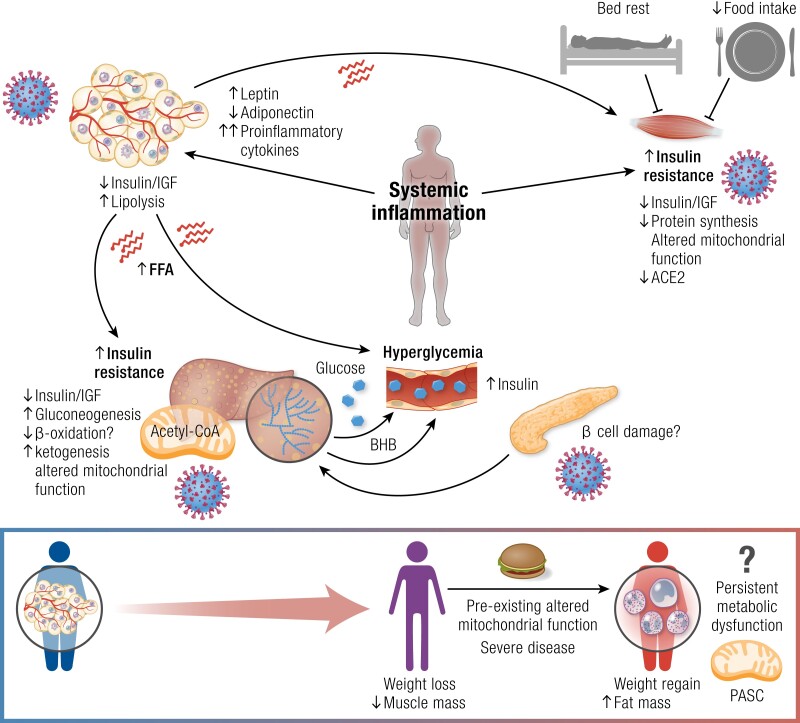
Schematic representation of the effects of SARS-CoV-2 on organs involved in energy metabolism. SARS-CoV-2 infects the adipose tissue, enhancing preexisting inflammation in people with obesity/diabetes, which may result in an uncontrolled inflammatory response. COVID-19 and inflammation lead to adipocyte dysfunction, causing multiorgan insulin resistance, which in skeletal muscle is also promoted by elevations in interferon gamma that impair the insulin/IGF pathway, and downregulation of the angiotensin-converting enzyme 2 (ACE2) receptor. In adipose tissue, insulin resistance and possibly endothelial dysfunction result in increased lipolysis. Free fatty acids (FFA) that are released into the circulation may further worsen insulin resistance and, in the liver, may lead to lipid accumulation and promote hepatic gluconeogenesis, which is also enhanced by hyperinsulinemia secondary to insulin resistance and SARS-CoV-2-induced Golgi protein 73 (GP73) production. Altered mitochondrial function in the liver may impair fatty acid β oxidation and ketogenesis, which increases with increased COVID-19 severity, but is blunted in comparison to acute respiratory distress syndrome resulting from influenza virus. Muscle disuse and disease-related malnutrition result in muscle mass loss and further impairment of glucose disposal. Hyperglycemia in patients with COVID-19 may result from these mechanisms, and it is likely that only individuals who are predisposed to impaired glucose metabolism, possibly because of preexisting metabolic dysfunction, will develop long-term hyperglycemia and metabolic dysfunction, with COVID-19 acting as a second hit. In these persons, especially those who had severe COVID-19, weight regain with preferential fat catch-up during recovery may further worsen metabolic health, leading to persistent metabolic dysfunction and post-acute sequelae of COVID-19 (PASC). Abbreviations: ACE2, angiotensin-converting enzyme 2; BHB, beta-hydroxybutyrate; FFA, free fatty acids.

The COVID-19 pandemic provides the opportunity to enhance our understanding of how metabolic alterations interact with acute inflammation to trigger immunoinflammatory and further dysregulation of energy metabolism, both during the acute phase and after recovery. During the acute phase, a strong inflammatory response shifts the metabolism toward catabolic pathways to provide substrates for energy production and support the immune response. These are mechanisms that have been well described in individuals with sepsis (194). However, SARS-CoV-2 appears to exert specific metabolic effects (Fig. 5). Infection of adipose tissue by the virus triggers adipose tissue dysfunction, inducing altered adipokine secretome and lipolysis, leading to an increase in FFA in the circulation (145) that may promote systemic insulin resistance (107). SARS-CoV-2-induced IFN-γ (295), and downregulation of ACE2 (296, 297) may also contribute to the development of systemic insulin resistance. The increase in circulating FFA, together with SARS-CoV-2-induced GP73 production (246), metabolic factors such as MPO (191), activation of phosphoenolpyruvate carboxykinase (206), and hyperinsulinemia in response to insulin resistance, stimulate hepatic gluconeogenesis. Thus, hyperglycemia in people with COVID-19 is mainly driven by insulin resistance (107, 191, 309), which may persist even after recovery (191, 275, 276, 279). Preexisting mitochondrial impairment, which is a feature of obesity and type 2 diabetes (349), might be a key mechanism that aggravates disease severity and contributes to PASC and long-lasting metabolic alterations in these populations. SARS-CoV-2 infection profoundly impacts mitochondrial structure and function (350). Mitochondria are key not only for energy production, but also for biosynthesis of fatty acids, regulation of cell cycle, apoptosis, innate immune response, and ketogenesis (85, 254, 351). Ketogenesis maximizes the production of energy from adipose-tissue-derived fatty acids, supports the immune response, and reduces inflammation (254). Despite increased levels of ketone bodies with increasing COVID-19 severity, an impairment in ketogenesis has been reported (264), suggesting reduced mitochondrial activity in the liver. There is also evidence of mitochondrial impairment in other organs, as indicated by elevations in growth differentiation factor 15, a marker of skeletal muscle bioenergetic dysfunction (269). These alterations may persist and underlie PASC (352). Accordingly, low fatty acid oxidation and altered lactate production in skeletal muscle during graded exercise has been reported in COVID-19 survivors, which might contribute to the functional limitation of individuals with PASC (353). Mitochondrial and metabolic alterations are also observed in individuals with critical illness myopathy (354), where mitochondrial biogenesis, dynamics, as well as fatty acid oxidation and NADH-linked respiration appear to be altered and to contribute to muscle atrophy (355-358). Normalization of metabolic processes seems to occur in individuals who recover, whereas metabolic derangements persist in those with worsening disease and those who develop PASC. Other factors, such as changes in body composition resulting from inactivity and changes in food intake (a decrease during illness and an increase during recovery) impact metabolic health and may lead to persistent alterations, increasing cardiometabolic risk. However, observations that the impact of COVID-19 on glycemic control of patients with type 2 diabetes (an increase of 0.09% in HbA1c levels) is statistically significant but clinically negligible (336) and that the impairment in glucose metabolism does not persist in all people (21) are somehow reassuring and suggest that the metabolic alterations observed during the acute phase of COVID-19 are transient in most individuals.

## Conclusions and Future Perspectives

COVID-19 can directly and indirectly impair energy metabolism, which may persist in some people. Most mechanistic studies only investigated the effect of acute, severe COVID-19, so that it cannot be excluded that mild and subclinical forms have only minor metabolic effects and consequences. During the pandemic, particularly during its early phase, an impressive amount of reports have been published, but often lacking rigorous methodology (359) and yielding contradictory or unreliable results. Furthermore, global data sharing and accessibility have been rather limited (360), which still leaves plenty of room for exploration and consolidation of current knowledge. Detailed mechanistic studies and longer term follow-up of PASC will tell us whether and to what extent metabolic alterations are persisting or fully reversible. Several strategies targeting the mechanisms involved in the development of severe disease and PASC appear promising. For example, people with COVID-19 treated with the lipid lowering drugs fibrates showed significantly lower biomarkers of immunoinflammation and faster recovery, with fenofibrate reversing lipid accumulation and blocking SARS-CoV-2 replication in vitro (229). Genetic ablation or pharmacological inhibition of the mitochondrial pyruvate carrier attenuated disease severity in mice with SARS-CoV-2 pneumonia, along with reduced blood glucose and hyperlipidemia following viral pneumonia in obese mice (361). Most recently, it has been reported that the antihyperglycemic drug metformin reduced the risk of long-COVID by approximately 40%, this reduction being greater when the drug was started within 3 days of symptom onset (362, 363). Weight loss reduced both NRP1, a co-receptor that facilitates the binding of SARS-CoV-2 to ACE2 (312) and ACE2 in subcutaneous adipose tissue of individuals with overweight/obesity (364), which could reduce the susceptibility of adipose tissue to SARS-CoV-2 (364). Ketogenic diets have emerged as an effective strategy for weight loss and glycemic control (365) that might also help improve immune function (366), and thereby represent a promising weight loss strategy in the context of COVID-19 prevention and management.

Regardless of the mechanisms linking COVID-19 and metabolic diseases, the COVID-19 pandemic has a dramatic potential for further rise of the diabetes pandemic. It is therefore of the utmost importance to perform active screening for metabolic diseases in individuals with a history of COVID-19, and to further investigate pathomechanisms and targeted treatment strategies for COVID-19-related metabolic diseases.

## Abbreviations

ACE2: angiotensin-converting enzyme-related carboxypeptidase
ALT: alanine aminotransferase
ARDS: acute respiratory distress syndrome
AST: aspartate aminotransferase
BMI: body mass index
ER: endoplasmic reticulum
FFA: free fatty acid
GP73: Golgi protein 73
GRP78: glucose-regulated protein 78
HbA1c: glycated hemoglobin
HOMA-IR: homeostasis model assessment of insulin resistance
HR: hazard ratio
IFN: interferon
IP-10: interferon-γ-induced protein-10
IQR: interquartile range
IRF1: interferon regulatory factor 1
MPO: myeloperoxidase
NAFLD: nonalcoholic fatty liver disease
NLRP3: NOD-like receptor pyrin-domain containing 3
NRP1: neuropilin 1
PASC: post-acute sequelae of COVID-19
PLA2: phospholipase A2
REST: RE1-silencing transcription factor
STING: stimulator of interferon genes
T_H_1: T helper 1
TMPRSS2: transmembrane protease serine 2
